# Biocompatible Nanocomposite Enhanced Osteogenic and Cementogenic Differentiation of Periodontal Ligament Stem Cells In Vitro for Periodontal Regeneration

**DOI:** 10.3390/ma13214951

**Published:** 2020-11-04

**Authors:** Jin Liu, Quan Dai, Michael D. Weir, Abraham Schneider, Charles Zhang, Gary D. Hack, Thomas W. Oates, Ke Zhang, Ang Li, Hockin H. K. Xu

**Affiliations:** 1 Key Laboratory of Shannxi Province for Craniofacial Precision Medicine Research, College of Stomatology, Xi’an Jiaotong University, Xi’an 710004, China; liujin8511@163.com (J.L.); daiquan06@126.com (Q.D.); 2 Clinical Research Center of Shannxi Province for Dental and Maxillofacial Diseases, College of Stomatology, Xi’an Jiaotong University, Xi’an 710004, China; 3 Department of Advanced Oral Sciences and Therapeutics, University of Maryland Dental School, Baltimore, MD 21201, USA; michael.weir@umaryland.edu (M.D.W.); charleszhang8@yahoo.com (C.Z.); GHack@umaryland.edu (G.D.H.); TOates@umaryland.edu (T.W.O.); 4 Department of Oncology and Diagnostic Sciences, University of Maryland School of Dentistry, Baltimore, MD 21201, USA; schneider66@umaryland.edu; 5 Member, Marlene and Stewart Greenebaum Cancer Center, University of Maryland School of Medicine, Baltimore, MD 21201, USA; 6 Department of Orthodontics, School of Stomatology, Capital Medical University, Beijing 100069, China; 7 Center for Stem Cell Biology & Regenerative Medicine, University of Maryland School of Medicine, Baltimore, MD 21201, USA

**Keywords:** biocompatible nanocomposite, osteogenic and cementogenic differentiation, periodontal ligament stem cells, periodontal regeneration

## Abstract

Decays in the roots of teeth is prevalent in seniors as people live longer and retain more of their teeth to an old age, especially in patients with periodontal disease and gingival recession. The objectives of this study were to develop a biocompatible nanocomposite with nano-sized calcium fluoride particles (Nano-CaF_2_), and to investigate for the first time the effects on osteogenic and cementogenic induction of periodontal ligament stem cells (hPDLSCs) from human donors.Nano-CaF_2_ particles with a mean particle size of 53 nm were produced via a spray-drying machine.Nano-CaF_2_ was mingled into the composite at 0%, 10%, 15% and 20% by mass. Flexural strength (160 ± 10) MPa, elastic modulus (11.0 ± 0.5) GPa, and hardness (0.58 ± 0.03) GPa for Nano-CaF_2_ composite exceeded those of a commercial dental composite (*p <* 0.05). Calcium (Ca) and fluoride (F) ions were released steadily from the composite. Osteogenic genes were elevated for hPDLSCs growing on 20% Nano-CaF_2_. Alkaline phosphatase (ALP) peaked at 14 days. Collagen type 1 (COL1), runt-related transcription factor 2 (RUNX2) and osteopontin (OPN) peaked at 21 days. Cementogenic genes were also enhanced on 20% Nano-CaF_2_ composite, promoting cementum adherence protein (CAP), cementum protein 1 (CEMP1) and bone sialoprotein (BSP) expressions (*p <* 0.05). At 7, 14 and 21 days, the ALP activity of hPDLSCs on 20% Nano-CaF_2_ composite was 57-fold, 78-fold, and 55-fold greater than those of control, respectively (*p <* 0.05). Bone mineral secretion by hPDLSCs on 20% Nano-CaF_2_ composite was 2-fold that of control (*p <* 0.05). In conclusion, the novel Nano-CaF_2_ composite was biocompatible and supported hPDLSCs. Nano-CaF_2_ composite is promising to fill tooth root cavities and release Ca and F ions to enhance osteogenic and cementogenic induction of hPDLSCs and promote periodontium regeneration.

## 1. Introduction

Periodontitis is a frequently-occurring infectious disease, manifested by gingival inflammation, eventually leading to damage to the periodontium which consists of alveolar bone, periodontal ligaments (PDL), cementum, and gingiva [1]. All over the world, one out of every two adults is affected by periodontal disease [2]. Furthermore, periodontal diseases are occurring at a rapidly growing rate [2]. When periodontal diseases progress, periodontium damage worsens and the patient eventually loses teeth, causing functional and aesthetic difficulties [3]. In addition to oral damage, periodontitis can also influence systemic diseases, including obesity, diabetes, cancer, cardiovascular diseases, and rheumatoid arthritis [4,5].

Common treatments for periodontal diseases include scaling and root planning. Flap surgery [6,7] can control acute inflammation, but the probability of gingival recession and root caries also increases, compromising the sustainability of the teeth after periodontal treatment. In addition, the older population has an elevated risk of root decay because of gingival recession and diminished saliva production [8,9]. Furthermore, the cementum coating covering the tooth roots is more susceptible to biofilm acid attacks than enamel and can be damaged or lost easily [10].

Indeed, root surface decays and elevated caries risk were present in about 10% of the patients with periodontal diseases [11]. Tooth decay in the roots is difficult to detect in the initial phase because of its obscure area. Tooth root decay can be clinically restored using a Class-V restoration. However, the restoration margins below the gingival line for root caries are difficult for hygiene and often become areas for periodontal microbial plaque growth. This in turn further aggravates the inflammation of the periodontium and gradually results in the destruction of periodontal attachment of the tooth [12].

In general, for the treatment of root caries, a dental restoration is needed when a cavity exists in tooth root [13]. It is meritorious for the filling materials to possess mineral-regeneration and antimicrobial abilities. Recent studies reported the development of bioactive dental polymers by using quaternary ammonium methacrylates (QAM), 12-methacryloyloxydodecylpyridinium bromide (MDPB), ethoxylated bisphenol A dimethacrylate (EBPADMA), and pyromellitic dianhydride glycerol dimethacrylate (PMGDM) [14]. A previous report demonstrated a composite containing metformin for filling deep cavities, which can induce the odontogenic induction and calcium production of dental pulp stem cells (DPSCs) [15]. Other novel multifunctional composites used nanoparticles of silver (NAg), 2-methacryloyloxyethyl phosphorylcholine (MPC), dimethyl-aminohexadecyl methacrylate (DMAHDM) and nanoparticles of amorphous calcium phosphate (NACP). These strategies achieved the suppression of metabolic activity, polysaccharide secretion and biofilm development of three types of periodontal pathogens [16].

Fluoride (F) is important in the suppression of dental caries, through the decrease of demineralization and increase of remineralization. The fluoride methods are useful for patients with lower salivary production or compromised oral hygiene ability [17]. Calcium (Ca) ions have shown importance in biological processes, including the entire lifecycle of bone from formation to muturation [18]. Researchers have evaluated the influence of stable Ca-based coatings in osseointegration [19], and the effects of free Ca ions on new bone formation [20]. A literature search revealed that, to date, a literature search showed no reported study on bioactive dental composite for root caries with Ca and F ions release to enhance the osteogenic and cementogenic differentiation of human periodontal ligament stem cells (hPDLSCs).

Recently, nanosized calcium fluoride particles (Nano-CaF_2_) were developed using a spray-drying machine. The composite released F and Ca ions for the formation of fluoroapatite and suppression of caries [21]. Submerging the samples in solutions of pH 4–7 produced similar strength values for the Nano-CaF_2_ nanocomposites, and greater strength than commercial F-releasing controls [22]. In addition, the Nano-CaF_2_ composite was intelligent as it substantially enhanced the F ion release at cariogenic low pH, when these ions would be critically necessary to prevent tooth decay [22]. These composites with high strength and great amounts of ion release have excellent potential for restorations to suppress secondary caries and prevent restoration cracks. Moreover, a novel nanocomposites containing DMAHDM, MPC, and Nano-CaF_2_ had a potent antibacterial function and great amounts of F and Ca ion release, which could be used to defeat dental biofilms and protect the teeth [23]. However, the effect of Ca and F ions from the composite in root caries restorations on the osteogenic and cementuogenic differentiation of hPDLSCs remains unknown.

Therefore, the objective of present project was to determine for the first time the effects of novel Nano-CaF_2_ composite on the viability, proliferation, and osteogenic and cementogenic induction of hPDLSCs. The following hypotheses were evaluated: (1) Nano-CaF_2_ containing composites would have good load-bearing capability that match those of the commercial control composite; (2) Ca and F ions could be released from the composite; (3) Nano-CaF_2_ composite would highly promote the osteogenic and cementogenic gene inductions, and the alkaline phosphatase (ALP) activity of hPDLSCs; (4) hPDLSCs on Nano-CaF_2_ composite would be able to synthesize significantly more bone minerals than that on commercial control composite.

The null hypotheses were: (1) Nano-CaF_2_ composite would have an inferior load-bearing capability compared to commercial control composite; (2) Nano-CaF_2_ composite would have little Ca and F ion release; (3) Nano-CaF_2_ composite would have little effect on osteogenic and cementogenic gene inductions of hPDLSCs; and (4) hPDLSCs on Nano-CaF_2_ composite would synthesize similar amounts of bone minerals to those on commercial control composite.

## 2. Material and Methods

### 2.1. Preparation of Composite Disks

The Nano-CaF_2_ was produced by employing a spray-drying machine as detailed previously [21]. Briefly, 0.10 g of CaF_2_ powder was suspended in 1 L of distilled water. The dilute suspension was sonicated for 2 h at 60 °C in an ultrasonic cleaner (3510R-MTH, Bransonic, Danbury, CT, USA), then pumped into a spray-drying machine (ViscoMist, Lechler, St. Charles, IL, USA) [24]. The CaF_2_ liquid was flowed to a spraying tube (ViscoMist) at a speed of 20 mL/min and atomized into a chamber with an elevated temperature (≈70 °C) of the spray-drying machine. The CaF_2_ nanoparticles in the air circulation were harvested by using an electrostatic precipitator (MistBuster, Air Quality Engineering, Minneapolis, MN, USA). The NH_4_OH was eliminated as NH_3_ and H_2_O vapors with the air circulation. Then the Nano-CaF_2_ was obtained at the electrostatic precipitator. The resulting nanopowder was verified as being CaF_2_ by X-ray diffraction [21]. Multipoint BET surface area evaluation of the particles were done (AUTOSORB-1, Quantachrome, Boynton Beach, FL, USA) with ultra-high-purity nitrogen being the adsorbing gas and liquid nitrogen being the cryogen. Transmission electron microscopy (TEM, 3010-HREM, JEOL, Peabody, MA, USA) was performed to evaluate the particle sizes in a previous study, which found that the mean particle size was 53 nm for the Nano-CaF_2_ particles [25].

Bisphenol glycidyl dimethacrylate (BisGMA, Esstech, Essington, PA, USA) and triethylene glycol dimethacrylate (TEGDMA, Esstech) were mixed at a mass ratio of 1:1 (all mass fractions, unless specified otherwise) [26,27,28]. Then, 0.2% camphorquinone (CQ, Esstech) and 0.8% ethyl 4-N, N-dimethylaminobenzoate (4E, Esstech) were added to enable the composite to be light-curable [29]. This resin matrix with BisGMA and TEGDMA is named as BT. For load-bearing capability, particles of barium boroaluminosilicate glass (Dentsply, Milford, CT, USA) with a median particle size of 1.4 μm were silanated using 4% 3-methacryloxypropyltrimethoxysilane (Esstech) together with 2% n-propylamine (Esstech) [30]. Then, Nano-CaF_2_ particles were mixed into the composite paste with groups described below.

In addition, Heliomolar (Ivoclar, Amherst, NY, USA) was employed as a clinical composite control. It consisted of 40–200 nm nano-sized silica with ytterbium-trifluoride using a filler amount of 66.7% by weight. Five groups were evaluated:

(1) 30% BT + 70% glass particles (0% Nano-CaF_2_ control);
(2) 30% BT + 60% glass particles + 10% Nano-CaF_2_ (10% Nano-CaF_2_);
(3) 30% BT + 55% glass particles + 15% Nano-CaF_2_ (15% Nano-CaF_2_);
(4) 30% BT + 50% glass particles + 20% Nano-CaF_2_ (20% Nano-CaF_2_);
(5) Commercial Heliomolar composite (Heliomolar control)

The filler mass fraction of 70% was blended to form the experimental composites. The samples for cell tests were made as described in a previous report [31]. Briefly, the cover of a sterile 96-well plate (Costar, Corning, NY, USA) was employed as the sample molds to form composite disks with nearly 8 mm in diameter and 1 mm in thickness. The disks were light-polymerized (Triad 2000, Dentsply, PA, USA) for 1 min [32]. The polymerized composite disks were submerged in distilled water at 37 °C and stirred for 1 day to eliminate any unpolymerized monomers, as described in a previous report [33]. Sterilization of the composites were achieved using ethylene oxide (AnproleneAN 74i, Andersen, Haw River, NC, USA). Samples were then degassed for 3 days before use, as described by procedures from the manufacturer [26].

### 2.2. Mechanical Testing

Flexural strength and elastic modulus of composites were determined in three-point flexure with a 20-mm span on a computer-controlled Universal Testing Machine (5500R, MTS, Cary, NC, USA) at a loading speed of 1 mm/min (n = 6). Flexural strength was evaluated: S = 3*F_max_ L*/(2*bh*^2^), where *F_max_* is the maximum load on the load-displacement (*F*-*d*) curve, *L* is the span, *b* is the specimen width and *h* is the thickness. Elastic modulus was evaluated: E = (*F*/*d*) (*L*^3^/[4*bh*^3^]) [34], where load *F* divided by displacement *d* is the slope of the load-displacement curve in the linear elastic region. The hardness values of composites were evaluated using a hardness tester (HMV-G 21DT, Shimadzu, Kyoto, Japan) with a Vickers diamond indenter. Three indents were performed and determinations were made at various locations on each sample with a 200 g force for 15 s of dwell time (n = 6) [35].

### 2.3. Measurement of Ca and F ion Release from Nano-CaF2 Composites

Specimens containing 10%, 15% and 20% Nano-CaF_2_ were used for release measurements as the composites contained Nano-CaF_2_ nanoparticles for Ca and F release. To measure this ion release, a NaCl (VWR Chemicals, LLC, Fountain Parkway, OH, USA) solution (133 mmol/L) buffered with 50 mmol/L HEPES (Thermo Fisher Scientific, Waltham, MA, USA) (pH = 7; 37 °C) was used to immerse the specimens. Following a prior report [36], 3 samples of 2 mm × 2 mm × 12 mm were submerged in 50 mL solution (total 9 samples for three tubes), producing a sample volume/solution of 2.9 mm^3^/mL. This was similar to a sample volume per solution of about 3.0 mm^3^/mL in a prior report [37]. The concentrations of F released from the samples were determined vs. submerging time: 1, 2, 4, 7, 14, 21, 28 and 56 days. At each time period, aliquots of 2 mL were taken for evaluation. The samples were taken to a fresh tube with new 50 mL NaCl of the solution. The amount of F ions was determined using a *F* ion-selective electrode, along with a reference electrode (Orion, Cambridge, MA, USA). The harvested solutions were diluted to a concentration to be inside the range of evaluation and then combined with an equal volume of a total ionic strength adjustment buffer (TISAB) solution (Fisher, Fair Lawn, NJ, USA). F ion standard solutions ranging from 1 × 10^6^ to 1 × 10^3^ mol/L were tested to develop a calibration plot, which was employed to measure the F ion concentrations. For Ca ions, the aliquots were measured for Ca ion concentrations by employing a spectrophotometric technique as described previously (DMS-80 UV-visible, Varian, Palo Alto, CA, USA) [38].

### 2.4. hPDLSC Culture

For the harvest of hPDLSCs, clinically healthy periodontal ligament PDL tissues were obtained from four premolars that were extracted from adult donors. The donors were 12–26 years of age and had their teeth removed due to orthodontic procedures [39]. The protocol was approved by the University of Maryland Baltimore Institutional Review Board (approval number: HP-00079029). hPDLSCs were obtained and characterized as described in prior reports with a minor modification [39,40]. Briefly, the PDL tissues were obtained from the middle third of tooth root surfaces, and digested in 3 mg/mL collagenase I (Worthington Biochem, Freehold, NJ, USA) and 4 mg/mL dispase (Roche, Mannheim, Germany) for 1 h at 37 °C in a humid environment with 5% CO_2_. Then PDL tissues from five teeth of different donors were placed together in culture dishes (Costar, Cambridge, MA, USA) with Dulbecco’s modified Eagle’s medium (DMEM, GIBCO BRL, Grand Island, NY, USA). This was supplemented with 20% fetal bovine serum (FBS, Invitrogen, Carlsbad, CA, USA), 1% penicillin/streptomycin (P.S, GIBCO BRL), and incubated at 37 °C with 5% carbon dioxide (CO_2_). At three-day intervals, the culture medium was refreshed, until the cells grew and proliferated. The cell colonies were formed after 7 days, which were digested to a single cell suspension using the filter paper (Whatman, TISCH Scientific, North Bend, OH, USA) with 0.25% Trypsin-EDTA (GIBCO BRL). The single cell was moved into 24 well plates (Costar) and culture dishes for enlarge cultivation. After 7–14 days, the culture was subconfluent and the cells were harvested by trypsinization. The cells were then cultured in a fresh medium. The hPDLSCs produced via this technique expressed surface markers characteristic of MSCs (STRO1) (Abcam, Cambridge, MA, USA) and were negative for typical hematopoietic (CD34) (Abcam) [39]. The 2-5th passage hPDLSCs were harvested for the tests described below. Each composite disk was put in a well of a 48-well plate (Costar) with culture medium, and immersed at 37 °C. After 3 h, the hPDLSCs were seeded with 1 mL of culture medium in each well, as described in the following sections.

### 2.5. Cell Viability Assay

To determine if mixing Nano-CaF_2_ into composite would damage the adherent hPDLSCs, cell viability on the composite disks with different proportion of Nano-CaF_2_ and Helimalor was investigated via a cell counting kit-8 (CCK-8, Endo Life Sciences, Farmingdale, NY, USA), following to the manufacturer’s protocol. CCK-8 was based on the water-soluble tetrazolium salt. The WST-8 reaction yielded an orange water-soluble formazan dye in an amount that was correlated to the amount of live cells. First, each well with a composite disk was seeded with 1 mL of hPDLSC at a cell density of 5000 cells/well. The medium was refreshed at 3 day intervals. Cell proliferation at 1, 4, 7, 14 and 21 days was determined via the cell counting kit. The composite disks with cells were washed with phosphate buffered saline (PBS, Quality Biological, Gaithersburg, MD, USA) and moved to a new 48-well plate (Costar); then, 200-μL CCK-8 dye was placed to a well. The samples were put into a CO_2_ incubator for 2 h. The live cell numbers measured using the absorbance of the orange-colored formazan at an optical density of 450 nm (OD_450_ nm) using a microplate reader (SpectraMax M5, Molecular Devices, Sunnyvale, CA, USA). Six disks were evaluated in each group for every prescribed time period.

### 2.6. Scanning Electron Microscopy

The composite disks with hPDLSCs cultured for 14 days were observed using scanning electron microscopy (SEM, Quanta 200, FEI, Hillsboro, OR, USA). The composite disk-hPDLSC constructs (n = 6) were fixed with 1% glutaraldehyde (Millipore) in PBS, dehydrated with a graded series of ethanol (30–100%), and rinsed with hexamethyldisilazane (Millipore). The constructs were sputter-coated using platinum and then evaluated with SEM.

### 2.7. Live/Dead Staining

Separate composite disks were seeded with cells and cultured for live/dead staining to evaluate the hPDLSCs on composites with different mass fraction of Nano-CaF_2_. At each time point (1, 4, 7, or 14 days), the composite disks were removed from the wells of the 48-well plate, washed with PBS, and submerged in a live/dead staining solution at 37 °C for 15 min (Sigma-Aldrich, McLean, VA, USA). The solution contained 2 μM of calcein AM and 2 μM of propidium iodide [40]. Then, the constructs were examined with an inverted fluorescence microscope (Eclipse TE-2000S, Nikon, Melville, NY, USA) connected to a digital camera. Three random positions of every sample were imaged, with 4 samples resulting in 12 pictures for each group at each time point. The live and dead cells were counted. The percentage of live cells was: P_live_ = N_live_/(N_live_ + N_dead_), where N_live_ = the number of live cells, and N_dead_ = the number of dead cells [41]. The live cell density (D_live_) was calculated: D_live_ = N_live_/A, where A is the area of the view field for N_live_.

### 2.8. Quantitative Real-Time PCR

Cells with 5 × 10^4^ cells/well were seeded on each composite disk in the 24-well plate. After waiting 24 h for the cells to attach to the composite surface, the medium was replaced by an osteogenic medium, which consisted of DMEME growth medium, 10% FBS plus 100 nm dexamethasone, 10 mm β-glycerophosphate, 0.05 mm ascorbic acid, and 10 nm 1ɑ,25-dihydroxyvitamin D3 (Sigma-Aldrich). After cultured for 1, 7, 14, 21 days, quantitative real-time reverse transcription polymerase chain reaction (qRT-PCR) was employed to determine the gene expressions of osteoblastic and cementoblastic in hPDLSCs after being cultured using different composite samples. The total RNA was harvested using a Trizol reagent (Sigma-Aldrich) following the protocol. The RNA was reverse transcribed into cDNA using a High-Capacity cDNA reverse transcription kit (Applied Biosystems, Foster City, CA, USA). The expressions of osteogenic differentiation genes markers, included ALP, collagen type 1 (COL1), runt-related transcription factor 2 (RUNX2), osteopotin (OPN), the genes for cementogenic differentiation were cementum attachment protein (CAP), cementum protein 1 (CEMP1) and bone Sialoprotein (BSP). These genes were evaluated with qPCR employing the SYBR Green PCR Master Mix (Applied Biosystems), as previously described [31,42]. The housekeeping gene GAPDH (Sigma-Aldrich) was employed as an internal control to normalize the expression amounts of various genes [43]. The sequences of human specific primers used for the amplification of the indicated genes were synthesized commercially (Sigma-Aldrich) and are listed in Table 1. qPCR data collection and analyses were done via an Applied Biosystems Prism 7000 Sequence Detection System. The relative expression was determined via the 2^−ΔΔ^^Ct^ method and normalized using the cycle threshold (C_t_) values of GAPDH. C_t_ values of control group at day 1 was used as the calibrator (n = 6).

### 2.9. ALP Activity

The hPDLSCs were seeded onto composite disks in 48-well plates at a density of 10^4^ cells/well [44]. The ALP activity was determined via a QuantiChrom ALP Assay Kit (BioAssay Systems, Cambridge, MA, USA) at 1, 7, 14 and 21 days. Briefly, composite disk-hPDLSC samples were rinsed with cold PBS. Adherent cells were digested and washed using PBS, then suspended again and stirred in 0.2% Triton-X100 lysis buffer for 30 min. The samples were then subjected to centrifugation at 1500 rpm for 5 min. Then, the ALP activity of the supernatant was determined with an ALP working solution. The solution had 200 μL of assay buffer, 5 μL of Mg acetate (final 5 mm), and 2 μL of pNPP solution substrate (10 mm), with a ratio of 20 μL sample supernatant/180 μL solution. After being mixed, the samples were measured with the absorbance at OD_405_ nm, and again after 4 min using a microplate reader (SpectraMax M5), as described in the protocol from the manufacturer. ALP activity was normalized using the protein amount [31]. The protein amount was determined via the Micro BCA Protein Assay (Thermo Scientific, Rockford, IL, USA), as described in the protocol from the manufacturer. Then, the cell lysis supernatants were mingled with the working reagent in the kit, which consisted of reagent A and reagent B (50:1, Reagent A:B), at a volume ratio of 1:50. The colorimetric samples were employed for the absorbance determination at OD_562_ nm with the microplate reader (SpectraMax M5). Standard curves were formed using albumin standard ampule (BSA) at concentrations of 0, 25, 125, 250, 500, 750, 1000, and 2000 μg/mL, which were employed to determine the related protein amounts (n = 6).

### 2.10. Alizarin Red Staining (ARS) of Bone Minerals Secreted by hPDLSCs

The hPDLSCs were seeded onto the composite disks in 48-well plates at 1 × 10^4^ cells/well [44] and cultured for 1, 7, 14, and 21 days in the osteogenic medium. Six samples were evaluated in every group at every prescribed time point for bone mineral production (n = 6). Then, the bone mineral secreted by the hPDLSCs on composites was examined in alizarin red staining (ARS, Millipore), as described in the protocol from the manufacturer. Briefly, the cells on composite disks were fixed with 4% paraformaldehyde (Sigma-Aldrich) for 30 min and stained for 30 min by 2% ARS solution, which could stain calcium substance secreted by hPDLSCs to become a dark red color [45]. Then, the ARS liquid was removed, composite samples were washed with PBS to eliminate any loose alizarin red. The samples were then imaged. For quantitative measurement, the ARS-stained hPDLSCs on composites were de-stained in 10% cetylpyridinium chloride (Sigma-Aldrich) for 15 min. The solutions were evaluated at OD_652_ nm with the microplate reader (SpectraMax M5). The data were obtained using folds of change, with the OD data of control group on day 1 being the reference.

### 2.11. Statistical Analysis

All tests were repeated three times at different times by the same operators. Statistical analyses were performed using SPSS 20.0 (SPAA, Chicago, IL, USA). Data were analyzed via two-way analyses of variance (ANOVA), followed by Tukey’s test as a post hoc comparison. Sample size was determined based on previous studies and statistical analyses. For example, for flexural strength, a difference was chosen as the mean flexural strength of one setting being different from the rest by 30 MPa. In the power analysis, with a significance level of 0.05, a power of 0.95, and a typical standard deviation of 10 MPa, planning of specimen numbers with the estimation approach required 5 replications. To be conservative, 6 repeats (n = 6) were performed for flexural strength. Sample size for other tests were determined similarly. All data are shown as mean ± standard deviation of the mean (mean ± SD). The probability level (*p*) was considered significant at *p* < 0.05.

## 3. Results

**Figure 1:**
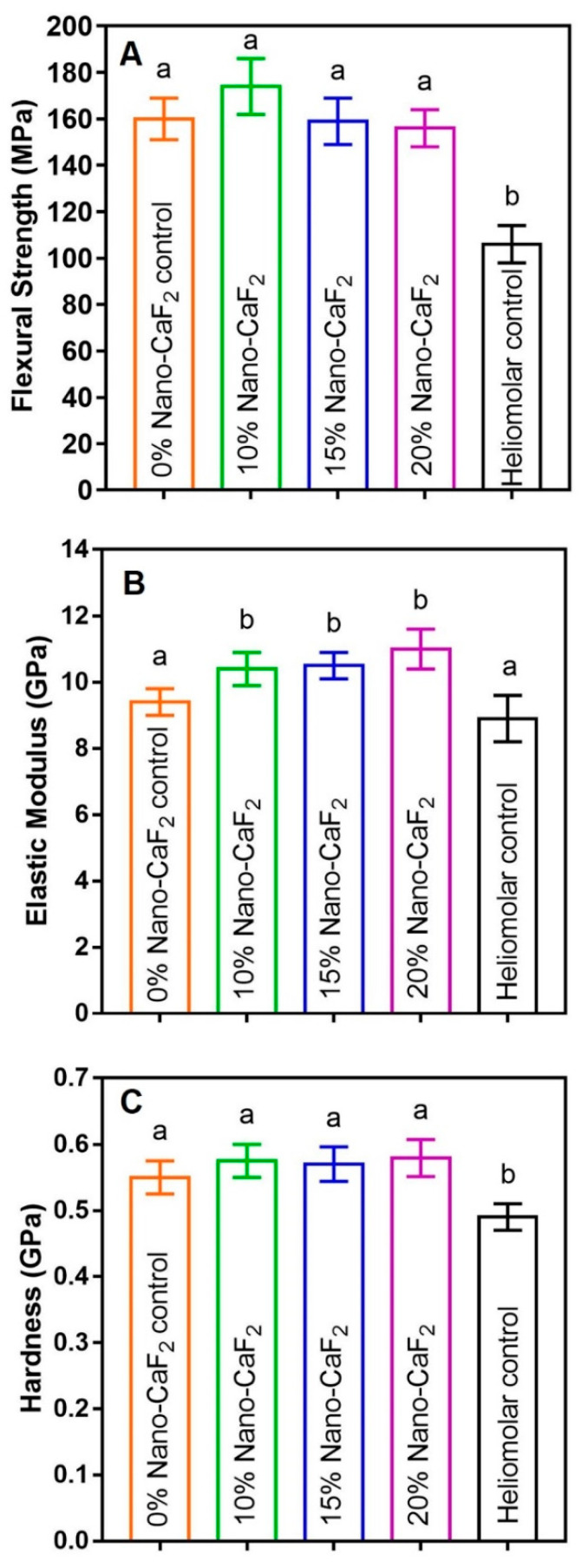
Flexural strength, elastic modulus and hardness of composites. (**A**,**C**) showed that there was no difference in flexural strength and hardness among the four Nano-CaF_2_ groups, but the flexural strength of Heliomolar was less than the others (*p* < 0.05). (**B**) The elastic moduli for 0% Nano-CaF_2_ and Heliomolar group were slightly less than other Nano-CaF_2_ groups. Values with dissimilar letters such as a and b are significantly different (*p* < 0.05, mean ± SD; n = 6).

Flexural strength, elastic modulus, and hardness were measured for the composites. Figure 1A,C (mean ± SD; n = 6) show that there was no difference in flexural strength and hardness among the four Nano-CaF_2_ groups. However, the flexural strength of Heliomolar control was only 2/3 of those of Nano-CaF_2_ groups (*p <* 0.05). The hardness of Heliomolar was 9/10 of those of Nano-CaF_2_ groups (*p <* 0.05). Figure 1B displays that 0% Nano-CaF_2_ and Heliomolar control had values that were 4/5 of those of other Nano-CaF_2_ groups (*p <* 0.05).

**Figure 2:**
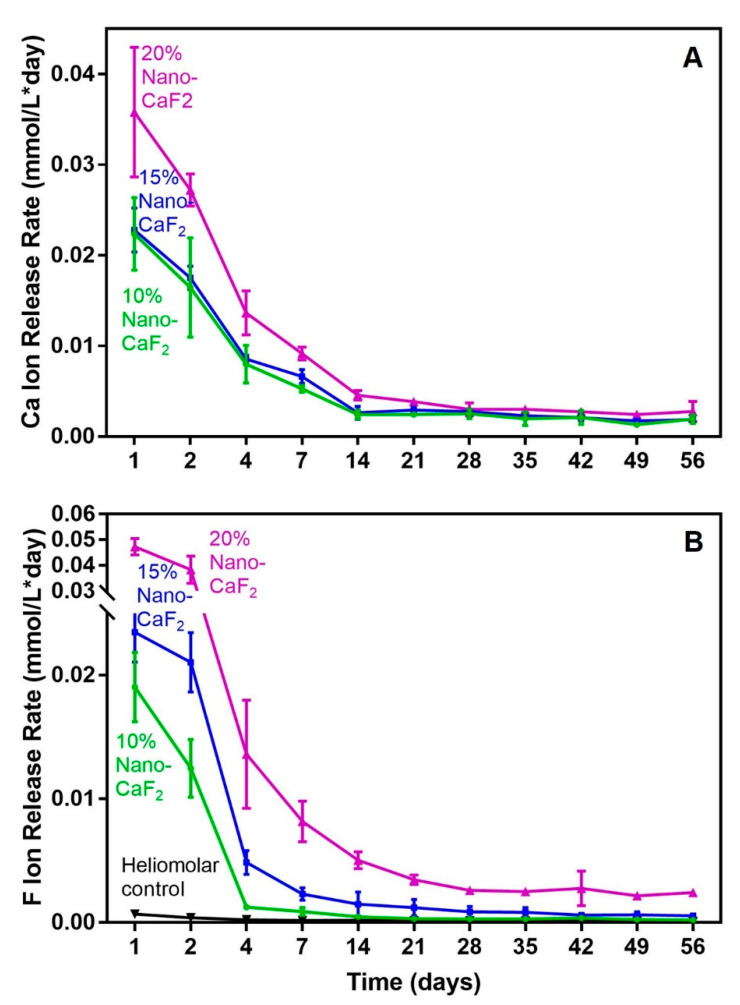
The release of Ca and F ions from CaF_2_ composites. (**A**) The Ca ions released from the 20% Nano-CaF_2_ was higher than the other composites initially. There was no difference among the three composites at the end time (*p* > 0.1). (**B**) The F ions released from the 20% Nano-CaF_2_ composite was higher than the other groups. The release from 20% Nano-CaF_2_ composite exceeded Heliomolar by nearly 30 folds (*p* < 0.05, mean ± SD; n = 6).

Figure 2 shows the ion release from the Nano-CaF_2_ composites (mean ± SD; n = 6). The 20% Nano-CaF_2_ composite had the highest release of Ca and F ions among all the tested groups. They gradually decreased from day 1 to day 14, and then remained stable. In particular, Heliomalar had no Ca ion release. The F ion release for 20% CaF_2_ composite was nearly 30 folds that of Heliomolar (Figure 2B).

**Figure 3:**
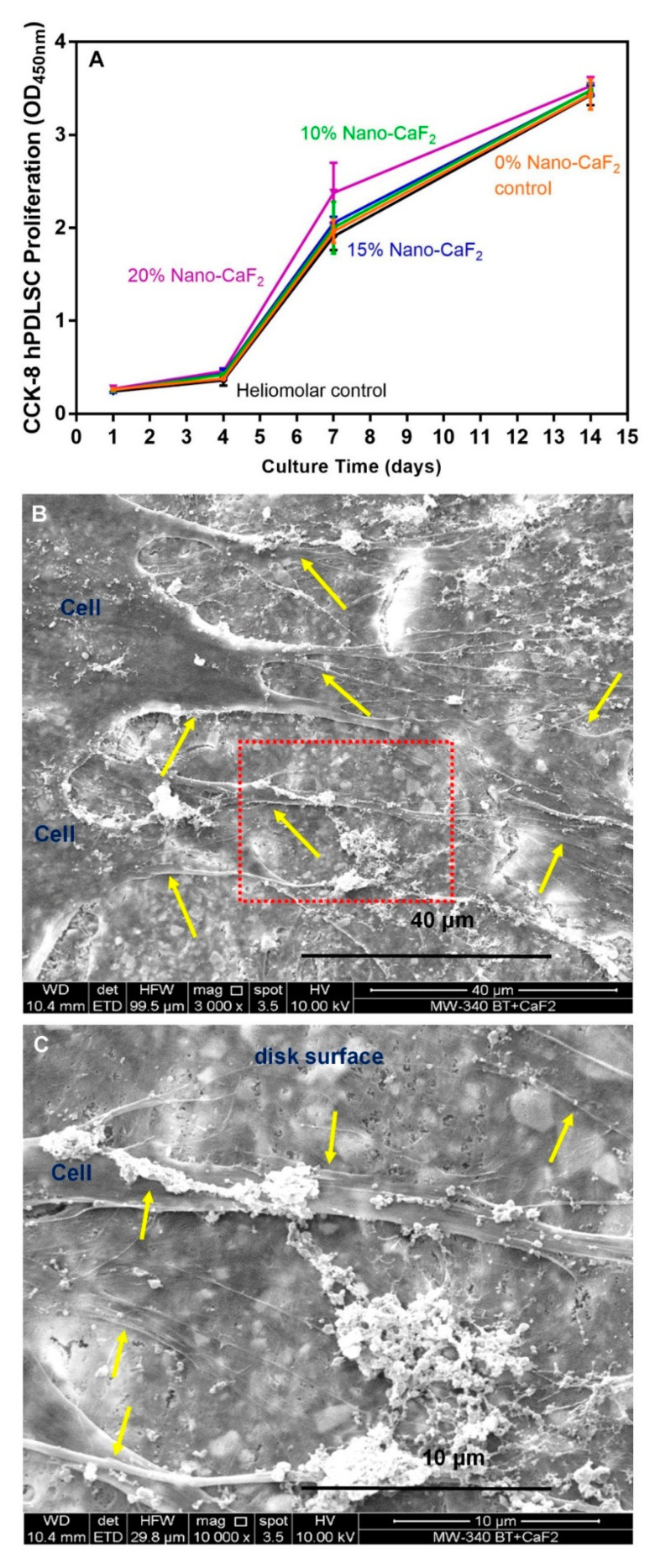
Proliferation and SEM images of hPDLSCs attaching to the composites. (**A**)The cell proliferation was not adversely affected by the addition of Nano-CaF_2._ The number of cells increased by approximately 12 folds from 1 to 14 days (*p* > 0.1, mean ± SD; n = 6). (**B**,**C**) The representative SEM images of hPDLSCs seeding on the surface of composite disks at 14 days. The hPDLSCs formed long cytoplasmic extensions (yellow arrows) that adhered to the surface of composite. Therefore, the composites were biocompatible and promoted hPDLSC adherence (n = 6).

Figure 3A shows that the cell proliferation was not adversely affected by the addition of Nano-CaF_2_ (mean ± SD; n = 6). The growth of the attached hPDLSCs on the composites with different proportions of Nano-CaF_2_ was similar to Heliomolar. Representative SEM images show hPDLSCs on composites at 14 days (Figure 3B). An enlarged picture of the red dotted frame is shown in Figure 3C. The hPDLSCs formed long cytoplasmic extensions (yellow arrows) on composites, exhibiting that the composite was biocompatible and promoted the hPDLSC adherence.

**Figure 4:**
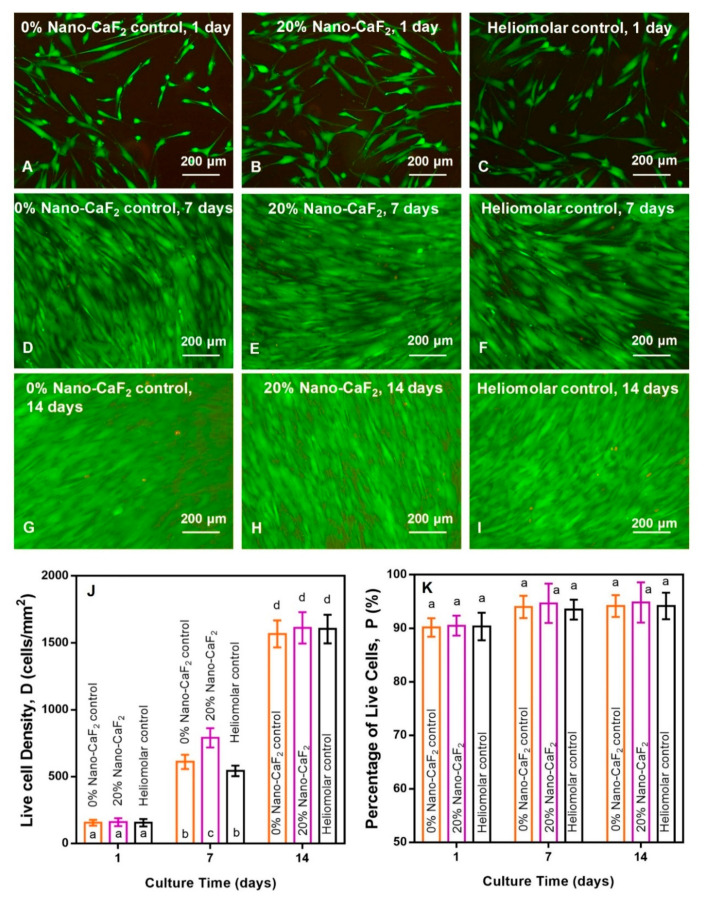
(**A**–**I**) The live/dead staining of hPDLSCs on omposites. Live cells (green) were numerous and dead cells were very few (n = 6). (**J**) The live cell density (the number of live cells per area) for 20% Nano-CaF_2_ composite was slightly higher than 0% Nano-CaF_2_ and Heliomolar control (by approximately 1.3 folds and 1.5 folds at 7 days) Values with dissimilar letters such as a and b are significantly different (*p* < 0.05). (**K**) The percentage of live cells was nearly constant in all three groups (*p* > 0.1, mean ± SD; n = 6).

Figure 4A–I display representative live/dead staining photos of hPDLSC growth on the composites. There were large numbers of live cells (green staining) and few dead cells (red staining) on Nano-CaF_2_ composites and Heliomolar. The live cell amount grew with culture time from 1 to 14 days. In Figure 4J (mean ± SD; n = 6), the live cell density for 20% Nano-CaF_2_ was slightly higher than that for 0% Nano-CaF_2_ and Heliomolar control at 7 days. Live cell density values for all groups were similar at 14 days. In Figure 4K, the percentage of live cells was nearly constant in all three groups from 1 to 14 days (*p* > 0.1).

**Figure 5:**
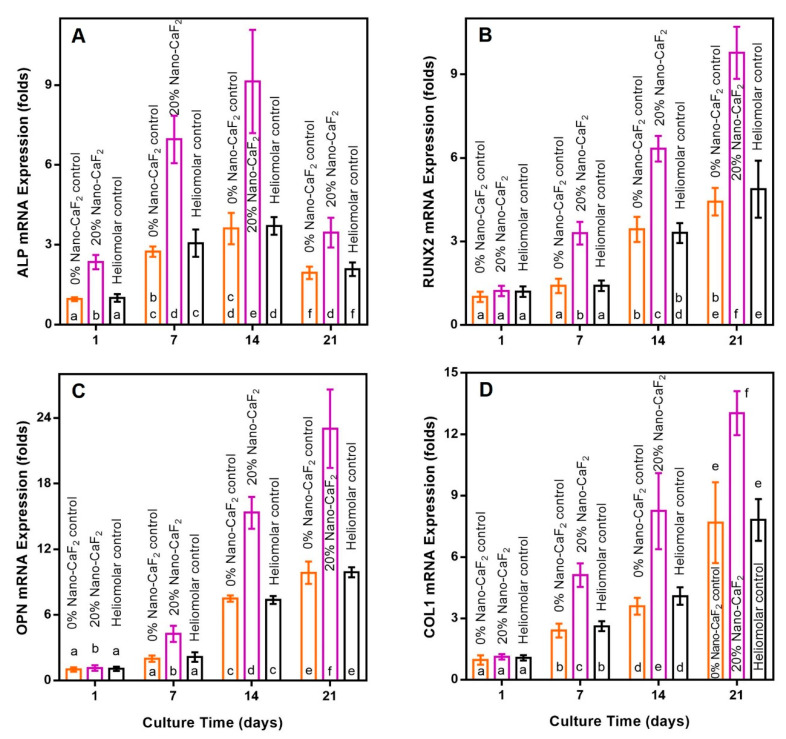
The expression of osteogenic genes of hPDLSCs growing on composites. The ALP gene expressions (**A**) peaked at 14 days, and RUNX2 (**B**), OPN (**C**) and COL1 (**D**) peaked at 21 days. Values with dissimilar letters such as a and b are significantly different (*p* < 0.05, mean ± SD; n = 6).

**Figure 6:**
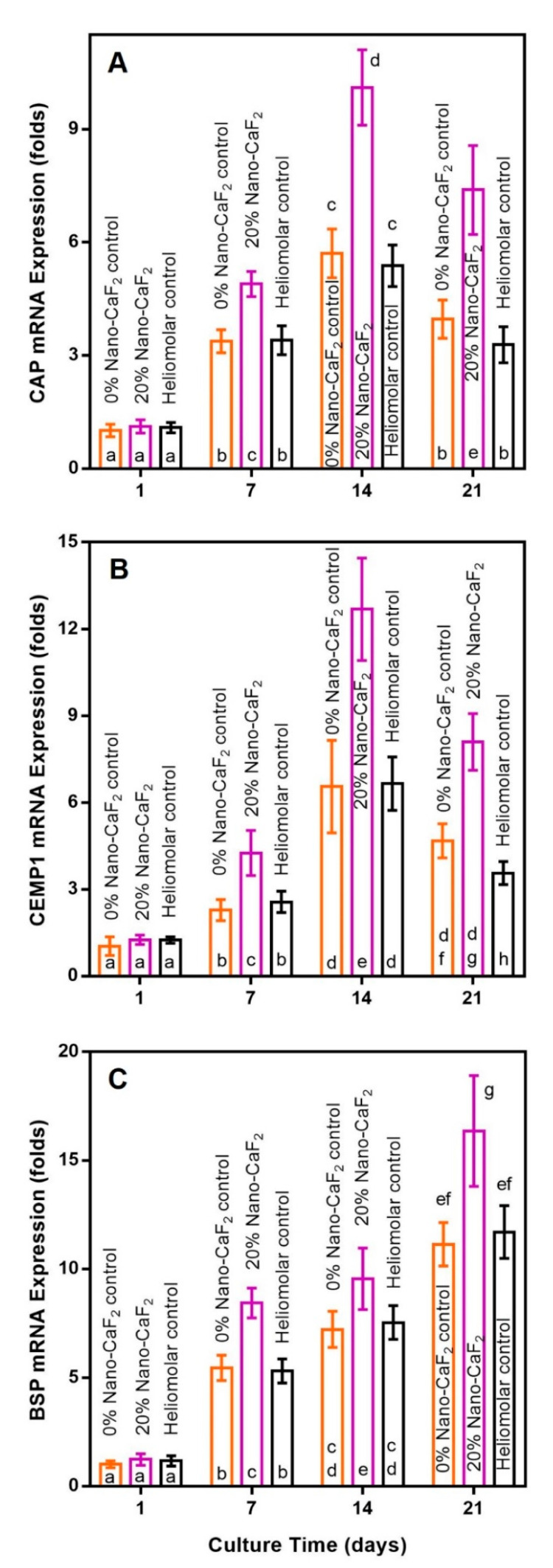
The expression of cementogenic genes of hPDLSCs growing on composites. All genes were promoted by the incorporation of 20% Nano-CaF_2_ in the composite at 14 days (CAP (**A**) and CEMP1 (**B**) genes) and 21 days (BSP gene (**C**). Values with dissimilar letters such as a and b are significantly different (*p* < 0.05, mean ± SD; n = 6).

For osteogenic genes (mean ± SD; n = 6), the ALP expression peaked at 14 days, and RUNX2, OPN and COL1 peaked at 21 days. The hPDLSCs on 20% Nano-CaF_2_ had significantly higher osteogenic gene expressions than the other two groups (Figure 5). The expressions of cementogenic genes were also promoted by the incorporation of 20% Nano-CaF_2_, as shown in Figure 6 (*p <* 0.05). Therefore, the 20% Nano-CaF_2_ composite promoted the osteogenic and cementogenic induction of hPDLSCs.

**Figure 7:**
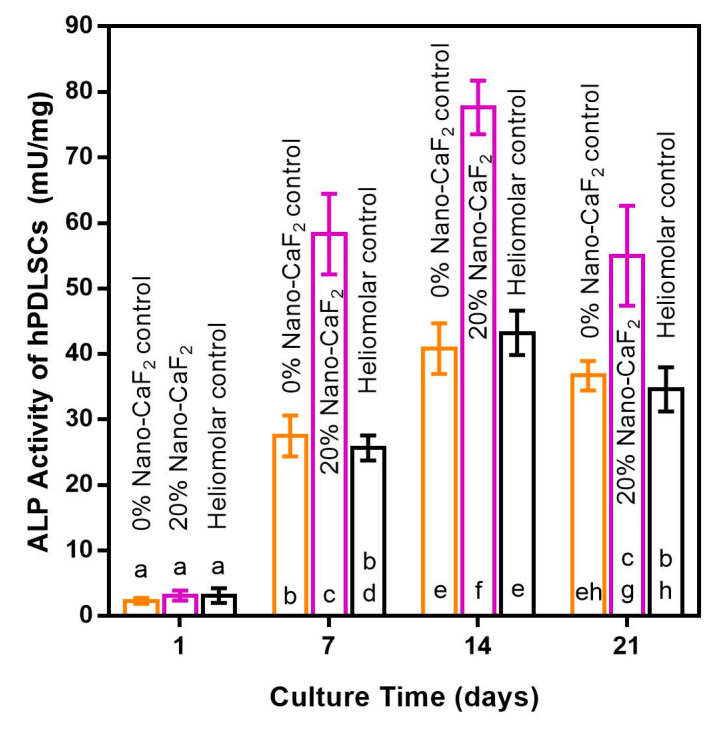
The ALP activity of hPDLSCs growing on composites. At 7, 14 and 21 days, the ALP activity of the 20% Nano-CaF_2_ group was 57-fold, 78-fold and 55-fold those of 0% Nano-CaF_2_ control at 1 day. Values with dissimilar letters such as a and b are significantly different (*p* < 0.05, mean ± SD; n = 6).

The ALP activity (mean ± SD; n = 6) of hPDLSCs increased with time from 1 to 14 days, then decreased from 14 to 21 days (Figure 7). At 7, 14 and 21 days, the ALP activity of hPDLSCs in the 20% Nano-CaF2 group was 57-fold, 78-fold and 55-fold, respectively, that of 0% Nano-CaF2 control at 1 day (*p* < 0.05).

**Figure 8:**
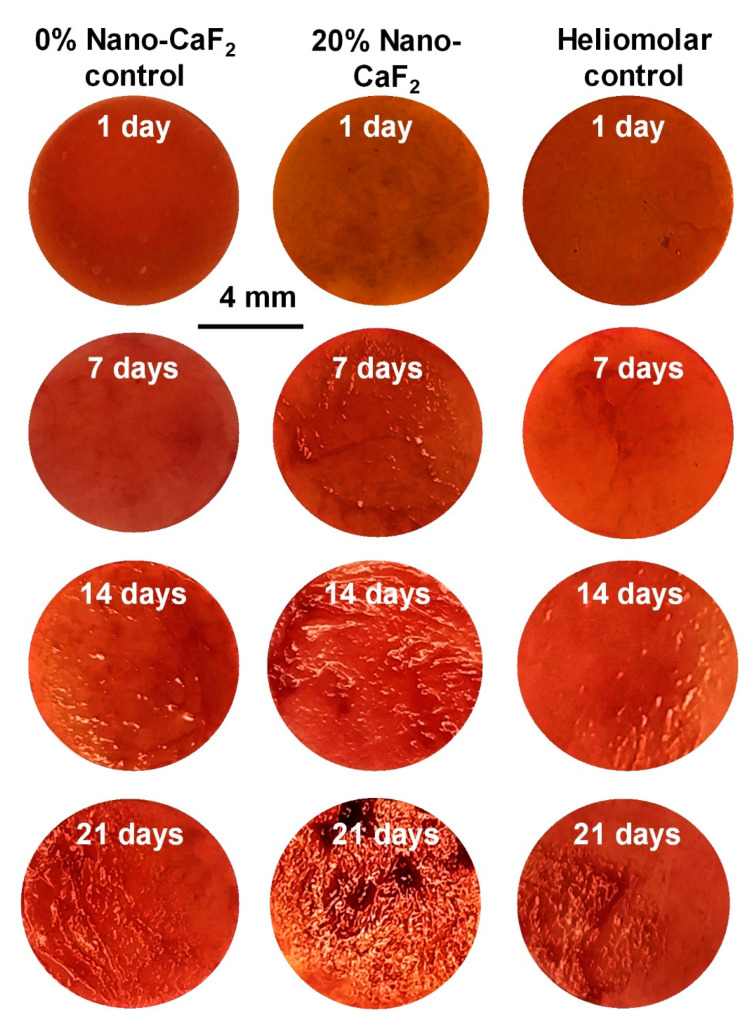
Representative ARS images of hPDLSC-synthesized bone mineral nodules (stained red). For 20% Nano-CaF_2_, bone mineral nodules started to appear at 7 days and increased at 14 days. The disks were covered by a layer of new mineralized bone matrix formed by hPDLSCs, which grew thicker with greater abundance at day 21. In contrast, there were much less mineral nodules on 0% Nano-CaF_2_ and Heliomolar control (n = 6).

**Figure 9:**
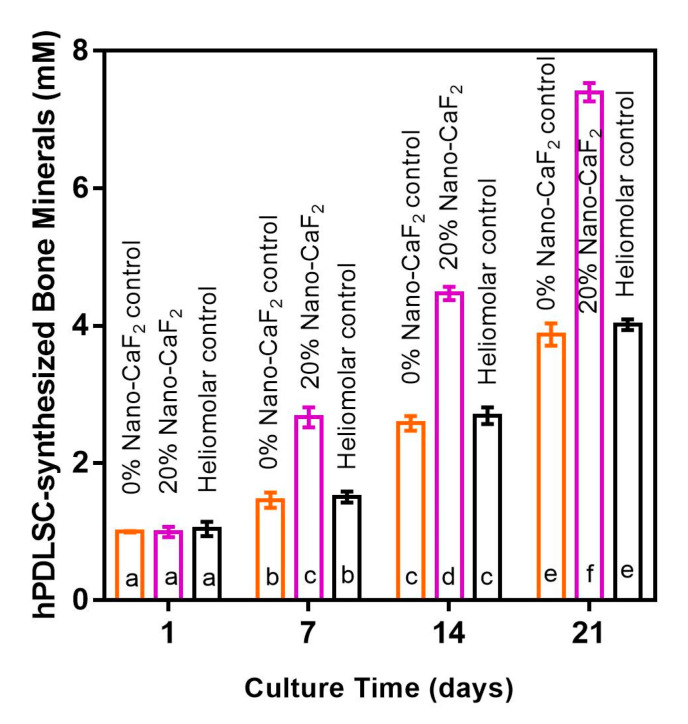
Quantitative bone mineral synthesis amounts by hPDLSCs on 20% Nano-CaF_2_ composite. The hPDLSC-synthesized bone minerals on 20% Nano-CaF_2_ composite at 14 and 21 days was nearly 2-fold those of the other groups (*p* < 0.05). Values with dissimilar letters are significantly different from each other. Values with dissimilar letters such as a and b are significantly different (*p* < 0.05, mean ± SD; n = 6).

Typical ARS pictures of hPDLSC-synthesized bone mineral nodules are shown in Figure 8. The bone minerals were stained red. There were no mineral nodules at 1 day in all groups. But for 20% Nano-CaF_2_, the hPDLSC started to synthesize bone mineral at 7 and 14 days. The composite disks were covered by a layer of new mineralized bone matrix secreted by the hPDLSCs, which grew thicker with greater abundance at 21 days. In contrast, there were much less bone mineral nodules on 0% Nano-CaF_2_ and Heliomolar control disks at 14 and 21 days. Figure 9 plots the quantitative bone mineral synthesis by hPDLSCs (mean ± SD; n = 6). The bone mineral secretion by hPDLSCs on 20% Nano-CaF_2_ composite substantially grew with longer culture time. The hPDLSC mineral secretion of 20% Nano-CaF_2_ group at 14 and 21 days was nearly 2-fold those of other groups (*p <* 0.05).

## 4. Discussion

The present study investigated the effects of novel Nano-CaF_2_ composite on the viability, proliferation, osteogenic, and cementogenic differentiation of hPDLSCs for the first time. The results showed that: (1) the new bioactive Nano-CaF_2_ composite had good mechanical properties that matched those of a traditional commercial control composite; (2) Ca and F ions were released steadily and continuously from the composite for up to one month; (3) the Nano-CaF_2_ composite produced good attachment and viability of hPDLSCs; (4) the 20% Nano-CaF_2_ composite substantially increased the expression of osteogenic and cementogenic genes and the ALP activity of hPDLSCs, compared to those at 0% Nano-CaF_2_ control and commercial composite control; (5) hPDLSCs on the 20% Nano-CaF_2_ composite synthesized much more bone mineral than that on 0% Nano-CaF_2_ and the commercial composite control. Therefore, the novel Nano-CaF_2_ composite is promising for tooth root cavities of periodontitis patients to not only fill the cavity, but also release Ca and F ions to enhance the osteogenic and cementogenic induction of hPDLSCs and regenerate periodontal tissues.

The hPDLSCs were used in the present study because they had the potential to differentiate into the ostegenic, fibrogenic and cementogenic lineages, which were suitable for periodontal regeneration. The bioactive Nano-CaF_2_ composite showed promise to promote periodontal regeneration via hPDLSCs, including alveolar bone and cementum. Further study should investigate gingival cells for gingival tissue regeneration [39].

For patients with periodontitis and gingival recession, the composite is not only a restorative material, but also a drug delivery vehicle for periodontal regeneration by releasing the active drug into the periodontal pocket. Ca ions can mediate platelet induction and provisional matrix synthesis, adhere to acidic-rich proteins, and produce supersaturating conditions for bone-mineral formation. High extracellular levels of Ca ions have been related to greater osteogenic cell activity and elevated levels of osteoclast apoptosis. Ca ions in the inorganic component of bone form a main component of hydroxyapatite. Ca ions partake in bone mineralization by producing supersaturating conditions via fixation by the Ca-binding proteins including glycosaminoglycans, and proteoglycans [46,47]. Therefore, this project provided a nanostructured composite for Class V filling applications that could release great amounts of Ca ions into the periodontal pocket to stimulate the hPDLSCs for periodontal regeneration.

Furthermore, in vitro and animal studies have demonstrated that F ions can control bone-forming cells and bone resorption, by influencing the RANKL/OPG system and directing the BMP/Smads signaling pathway or suppressing the NFATc1 gene expression to inhibit the osteoclastic activity [48]. In addition, local delivery of F ions had the ability to suppress tooth lesions and increase remineralization, thus increase the mineral density and help treatments for osteoporosis [49]. Therefore, the unique class of biomaterials containing Ca, F and P ions are highly meritorious for hard tissue repair and regeneration because of their superior biocompatible nature and bioactive abilities [24,50,51]. Then the release rate of Ca and F ions from different concentration of CaF_2_ composite showed that the 20% Nano-CaF_2_ had a higher release rate than the other groups initially.

Recently, nanoparticles with Ca, F and P ions were developed and incorporated into dental composites, showing good mechanical properties [36,52,53,54]. Load-bearing capabilities are important for dental composites to support biting stresses in the mouth. In the present study, the flexural strength, elastic modulus, and hardness of composites containing 10% and 20% CaF_2_ exceeded those of a commercial composite that has been used in patients. This indicates that the novel bioactive Nano-CaF_2_ composite has sufficient load-bearing properties to be used clinically where the commercial composite has been used, including Class V restorations. 

The growth of the attached hPDLSCs on the composite with different concentrations of Nano-CaF_2_ was similar to the Heliomolar control composite. Therefore, the live cell density and growth were not negatively influenced by the incorporation of the Nano-CaF_2_ ingredient. And the composites with or without Nano-CaF_2_ and the Heliomolar group all demonstrated excellent cell properties, enabled cell adherence, and supported cell growth. So the addition of Nano-CaF_2_ into the composite did not harm the hPDLSC growth, proliferation, and attachment, exhibiting an excellent cell compatibility.

The release of Ca, F and P ions was shown to induce remineralization for tooth enamel and dentin in previously studies [55]. Ca ions indeed helped induce cell differentiation in situ [56]. The induction of osteogenic genes was greatly promoted through the Ca-sensing receptor and the type L voltage-gated Ca ion channels [57]. In addition, the F ions regulate the cell proliferation and differentiation in two directions. F ions can stimulate cell proliferation at a low concentration (10^−7^–10^−5^ mol/L) and inhibit cell proliferation at a high concentration (10^−4^–10^−3^ mol/L) [58]. In the current project, the initial F ions release from the 20% CaF_2_ composite was the greatest among all the investigated groups. Nonetheless, it was only 0.047 mmol/L, which was equivalent to 4.7 × 10^−5^ mol/L. It then decreased to 0.008 mmol/L (=8 × 10^−6^ mol/L) at 7 days and 0.0025 mmol/L (=2.5 × 10^−6^ mol/L). Therefore, these low concentration of F ions in the present study exhibited no toxic influence for the hPDLSCs. 

Furthermore, low concentration of F ions can increase the activity and proliferation of osteoblast-like cells, increase the ALP activity and promote bone mineralization [59,60]. F ions can enhance the magnitude and opening of K^+^ selective ion channels [61]. This mechanism relies on the extracellular Ca ions and can be impeded by the Ca ion channel blockers [62]. This suggests that the early-stages in the responses of osteocytes to the F ions were likely organized by the cascade reaction of Ca ions as a second messenger or through the activity of K^+^ selective channels [61]. In the present study, the Ca and F ions in the composite displayed a continuous release for 4 weeks. From the results of the current project, 20% CaF_2_ composite was selected to be the optimal composite with great amounts of ion releases and good load-bearing characteristics. This composite is promising to be a delivery carrier to release the therapeutic ions into the diseased periodontal pocket, and there are methods to recharge the composite to achieve long-term ion release [63].

The Nano-CaF_2_ composite could delivery F ions to be locally released to stimulate hPDLSCs. Regarding the osteogenic, cementogenic, and mineralization-related gene expressions, several genes were promoted in the initial phase, and several genes were promoted during the later phase of osteogenic and cementogenic induction and mineral secretion process. The ALP, CAP and CEMP1 reached maximum at 14 days. RUNX2, OPN, COL1 and BSP reached maximum at 21 days. ALP protein secretion was also strongly enhanced at 14 days. Moreover, the bone mineral synthesis by hPDLSCs was promoted at 21 days, in agreement with this happening in the later stage of the cell induction progress. The effect of Heliomolar composite on the differentiation of hPDLSCs was weak, probably because of the very low level of F ions and without the release of Ca ions.

There are several advantages of the novel bioactive Nano-CaF_2_ composite when used as a restorative dental material. First, both Ca and F ions are released locally from the Nano-CaF_2_ composite. Other biomaterials, including glass ionomer cements, release only F ions. Second, the composite could be used to fill the root caries in periodontal pockets to promote hPDLSCs with enhanced osteogenic and cementogenic differentiation, which is beneficial to bone and cementum regeneration. Third, the Nano-CaF_2_ composite restoration could potentially also promote the proliferation and differentiation of hDPSCs for dentin regeneration, which warrants further investigation. This study has several limitations. First, the Nano-CaF_2_ composite was not antibacterial. Further study should incorporation antibacterial component to combat periodontal infections. Second, this study was limited to Ca and F ions, without testing the delivery of other bioactive agents and growth factors. Third, the experiments were performed in vitro; in vivo animal study is still needed.

## 5. Conclusions

This study developed a novel biocompatible nanocomposite that supported the hPDLSC viability, proliferation, and osteogenic and cementogenic differentiation for the first time. The Nano-CaF_2_ composite had good mechanical properties that exceeded those of a commercial control composite. High levels of Ca and F ions releases from Nano-CaF_2_ composite were achieved. The 20% Nano-CaF_2_ composite substantially increased the hPDLSC expressions of osteogenic and cementogenic genes. The 20% Nano-CaF_2_ composite had high levels of ALP activity, with synthesis of bone minerals by hPDLSCs that was nearly 100% greater than that via 0% Nano-CaF_2_ composite and commercial composite control. The biocompatible Nano-CaF_2_ composite is promising for tooth restorations, including root cavities of periodontitis patients, to release Ca and F ions to enhance the osteogenic and cementogenic differentiation of hPDLSCs and promote periodontal regeneration.

## Figures and Tables

**Table 1:** List of primer sequences used in Real time PCR.

Gene	Primers(5′-3′)
GAPDH	(F) GCACCGTCAAGGCTGAGAAC (R) ATGGTGGTGAAGACGCCAGT
ALP	(F) TCAGAAGCTAACACCAACG (R) TTGTACGTCTTGGAGAGGGC
RUNX2	(F) TCTGGCCTTCCACTCTCAGT (R) GACTGGCGGGGTGTAAGTAA
COL1	(F) CTGACCTTCCTGCGCCTGATGTCC (R) GTCTGGGGCACCAACGTCCAAGGG
OPN	(F) TCACCTGTGCCATACCAGTTAA (R) TGAGATGGGTCAGGGTTTAGC
CAP	(F) CCTGGCTCACCTTCTACGAC (R) CCTCAAGCAAGGCAAATGTC
CEMP1	(F) GGGCACATCAAGCACTGACAG (R) CCCTTAGGAAGTGGCTGTCCAG
BSP	(F) GAACCACTTCCCCACCTTTT (R) TCTGACCATCATAGCCATCG
